# Understanding the socio-economic and sexual behavioural correlates of male circumcision across eleven voluntary medical male circumcision priority countries in southeastern Africa

**DOI:** 10.1186/s12889-015-2135-1

**Published:** 2015-08-22

**Authors:** Fiona K. Lau, Sylvia Jayakumar, Sema K. Sgaier

**Affiliations:** Integrated Delivery, Global Development Program, Bill & Melinda Gates Foundation, Seattle, WA USA; Department of Global Health, University of Washington, Seattle, WA USA

## Abstract

**Background:**

Male circumcision (MC) has been demonstrated to be effective and cost-effective for HIV/AIDS prevention. Global guidance to adopt this intervention was announced in 2007 for countries with high HIV/AIDS prevalence and low MC prevalence. However, scale up of voluntary medical male circumcision (VMMC) programs in MC priority countries have been slow. Many of these countries have particular cultural barriers that impede uptake of this effective intervention. This analysis explored correlates of MC status among men and their socio-economic, health and sexual behaviour factors using DHS data (2006–2011) from 11 MC priority countries.

**Methods:**

Our analysis included univariate unadjusted analyses for individual countries and the region (by combining all countries into one dataset) and a multiple logistic regression model.

**Results:**

Individual country results vary widely but alignment was mostly found between unadjusted analyses and multiple logistic regression model. The model found that men who are of the Muslim faith, reside in urban areas, have higher or secondary education attainment, hold professional occupations, and be in the richest wealth quintile are more likely to be circumcised. Circumcision is also positively correlated with lower reports of STIs, safe sexual behaviour, and HIV/AIDS prevention knowledge.

**Conclusions:**

Since the data collected predate VMMC program launch in these countries, results can only indicate baseline associations. However, characteristics of these existing circumcision practices may be utilized for better population targeting and program management to achieve higher impact with this effective prevention strategy.

**Electronic supplementary material:**

The online version of this article (doi:10.1186/s12889-015-2135-1) contains supplementary material, which is available to authorized users.

## Background

According to the most current Joint United Nations Programme on HIV/AIDS (UNAIDS) report, worldwide HIV prevalence in 2013 is estimated to be 35 million people living with HIV where 24.7 million (or 71 %) reside in sub-Saharan Africa (SSA) [1]. By 2013, new infections are estimated to be 2.1 million world-wide with 1.5 million in SSA alone [1]. Although HIV incidence has decreased by 38 % from 2001, the number of people living with AIDS continues to increase as mortality rate declines from improved access to antiretroviral therapy (ART) [1]. Prevention services are essential in order to reduce new infections that adds to the expanding population living with this disease.

Epidemiologically, voluntary medical male circumcision (VMMC) is considered the most efficacious and cost effective intervention in reducing female to male HIV transmission in SSA where heterosexual transmission is the predominant mode of infection [2, 3]. Ample evidence have been available since the late 1980s which included observational studies (cross-sectional, case control and cohorts) and randomized control trials [3, 4]. The most striking evidence came from three randomized controlled trials (RCTs) from South Africa, Kenya, and Uganda where the reduction of HIV infection in men was found to be 55–61 % [5–7]. As a result, the World Health Organization (WHO) and UNAIDS recommended the scale up of VMMC in 14 priority countries where HIV prevalence is high and male circumcision (MC) prevalence is low [8]. The Demographic and Health Surveys (DHS) started including questions on MC status in 2003. Self-reported MC status varies greatly across different countries and also within traditionally circumcising countries. Two published studies used DHS data to perform cross-sectional analyses that explored the correlations between self-reported MC status, HIV/AIDS infection, STI, and several socioeconomic factors [9, 10]. Gebremedhin had shown significant HIV risk for men who were uncircumcised having adjusted for socio-demographic variables and sexual history [9]. Tram et al. explored the associations of MC with age, education, residence, wealth, religion, ethnicity and region. Age, religion, and ethnicity were found to be strong predictors of circumcision while education, wealth, and residence were not [10]. These results were not surprising as DHS surveys in these countries were performed prior to VMMC program scale-up, traditional circumcisions were expected to dominate for those self-reported to be circumcised.

In this study, we explored additional correlates of male circumcision status and model different predictors for 11 MC priority countries. We included demographic and socioeconomic characteristics (region, age, age at circumcision, residence, marital status, education, religion, wealth quintile, occupation and access to media), attitudes towards wife amongst married men, non-sexual (tobacco use) and sexual health attributes (alcohol consumption at last sex, safe sexual behavior, total lifetime sexual partners, current number of wives or partners, age at first sex, self-reported STI symptoms in the last 12 months and HIV prevention knowledge). We chose a variety of variables that might influence or be influenced by men’s circumcision status. We also hope that finding of this background circumcision analysis would facilitate future analyses in order to understand the impact of scale-up and provide insights on programming and policy decisions. For individual countries, we have carried out univariate analyses using these characteristics. We have also combined all countries for regional trends by using univariate analysis and constructing a multiple logistic regression model.

## Methods

The data used in this study was extracted from the Men’s questionnaire of the Demographic and Health Surveys (DHS) from 11 MC priority countries in Eastern and Southern Africa. This data is openly available via the DHS website. No human subject research was involved. Analysis was performed for men only. We have selected the most recent Standard DHS that are publically available on the DHS website for the following countries: Ethiopia (2011), Kenya (2008), Lesotho (2009), Malawi (2010), Mozambique (2011), Namibia (2006), Rwanda (2010), Swaziland (2006), Tanzania (2010), Zambia (2007), Zimbabwe (2011). Botswana, Uganda, and South Africa were excluded from the analysis since the male questionnaire is not available for Uganda and South Africa and access to data for Botswana is restricted. Each questionnaire provides a nationally representative sample of men age 15–49 or 15–59 years. The dependent variable was self-reported MC status (binary outcome as Yes or No) which did not distinguish circumcisions as performed by traditional or medical practitioners. Independent variables include: Age (five-year age groups), Place of residence (Urban, Rural), Marital status (Never in union, Married, Separated where Separated includes those widowed, divorced or no longer co-habiting), Education attainment (No education, Primary, Secondary, Higher), Religion (Muslim, Christian, others), Wealth Index (Poorest, Poorer, Middle, Richer, Richest), Occupation (Professional, Clerical, Skilled agricultural, Elementary worker, Non worker), Access to media is derived from frequency of reading newspaper, listening radio or watching television (No access, Poor, Fair, Good), Tobacco use (Yes, No), Attitude towards wife (Good attitude, Poor attitude), and HIV/AIDS prevention knowledge (Yes, No). Sexual behaviour variables include: Alcohol consumption at last sex (Yes, No), Safe Sexual Behavior (Yes, No), Total lifetime sexual partners (1, 2, 3+), Number of wives/partners currently (1, >1), Age at first sex (five-year age groups), and Presence of STDs/STIs during the last preceding 12 months (Yes, No). More information on combined variables is available in Additional Information (in Additional file 1: Table A1). We have chosen these variables with the view that they may be related to beliefs or behaviours that may influence circumcision uptake during program scale-up.

### Statistical analysis

Analyses were performed using STATA statistical package, version 10.0 software. Individual country analysis was performed as univariate or unadjusted logistic regression to explore associations between MC status and socio-demographic and sexual behavior variables. Regional analysis was performed by combining the 11 country datasets in univariate logistic regression as well. Factors that were found to be statistically significant (i.e. with *p*-value < 0.05) in the regional univariate analysis were included in the multiple logistic regression model with all potentially important co-variates adjusted for confounding [11]. We calculated adjusted odds ratios (ORs) and 95 % confidence intervals (CIs) for all variables. Two variables were excluded from the model due to lack of significant correlations in the univariate analyses: Attitude towards wife and Number of wives/partners currently. Alcohol consumption at last sex is also removed from the model because nearly 50 % of the data was missing.

## Results

**Fig. 1:**
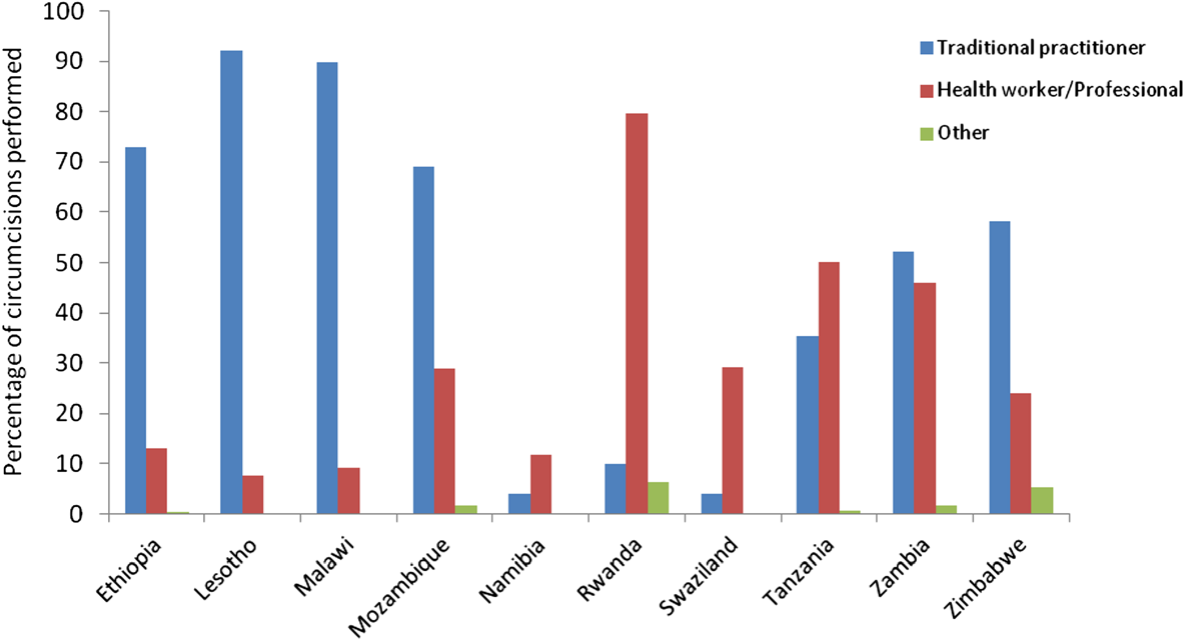
Distribution of circumcisions performed by country. Percentage of circumcisions performed by traditional practitioner (or family or friend), health worker (or medical professional), and others

**Fig. 2:**
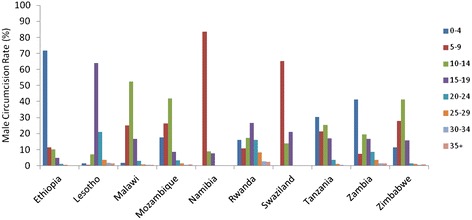
Rates of age at circumcision by country, by five-year age groups. Percentage of male circumcision performed grouped by age in five-year groups

For the 11 countries under study, results of univariate analyses of age distribution, education, wealth quintile, religious affiliation and urban/rural residence are already published by Tram et al. and therefore are not shown here but can be found in Additional Information (in Additional file 2: Table A2) [10]. Ethnicity was not included in this analysis as we aim to examine macro-level determinants to maximize generalizability. In terms of age, our analysis divided participants into five-year age groups. Similar to findings from Tram et al., older age groups showed higher likelihood of being circumcised. In fact, six out of the 11 countries analyzed showed that those who are 20 years or older have higher ORs for circumcision when compared to 15–19 year olds. Similar finding is also shown in the combined univariate analysis where all country data were merged into one dataset (Table 1). This is not surprising for baseline circumcision data where traditional circumcisions usually take place during adolescence; hence, as men get older more will have become circumcised. Circumcisions performed as reported by participants in these surveys showed a bimodal distribution with a majority of circumcisions (65 %) performed by traditional practitioners while others showed large proportions being performed by health worker or medical professionals (Fig. 1). Examining the distribution of age at circumcision (Fig. 2), it seems that countries with large proportions of circumcisions performed by medical professionals (i.e., Namibia, Rwanda, Swaziland, Tanzania and Zambia) also show age at circumcision being performed before age ten. The exact cause of this distribution is unclear as the surveys took place before the VMMC program was launched.

**Table 1:** Odds ratio of male circumcision status and socio-economic and behavioural characteristics with data combined for 11 countries

Characteristic	OR (95 % CI)	*p*-value
Age groups, years		
15–19	(Referent)	
20–24	1.37 (1.30–1.44)	0.000*
25–29	1.41 (1.33–1.48)	0.000*
30–34	1.44 (1.36–1.52)	0.000*
35–39	1.64 (1.54–1.74)	0.000*
40–44	1.65 (1.54–1.76)	0.000*
45–49	1.66 (1.54–1.78)	0.000*
50–54	1.64 (1.51–1.78)	0.000*
55–59	2.36 (2.12–2.63)	0.000*
Place of residence		
Urban	(Referent)	
Rural	0.83 (0.80–0.86)	0.000*
Marital status^a^		
Never in union	(Referent)	
Married	1.37 (1.33–1.42)	0.000*
Separate	1.39 (1.28–1.50)	0.000*
Education		
Higher	(Referent)	
Secondary	0.36 (0.33–0.38)	0.000*
Primary	0.62 (0.58–0.66)	0.000*
No education	1.75 (1.62–1.89)	0.000*
Religion^b^		
Muslim	(Referent)	
Christian	0.023 (0.021–0.026)	0.000*
Others	0.013 (0.011–0.014)	0.000*
Wealth Index		
Richest	(Referent)	
Richer	0.60 (0.58–0.63)	0.000*
Middle	0.62 (0.59–0.65)	0.000*
Poorer	0.67 (0.64–0.71)	0.000*
Poorest	0.73 (0.69–0.77)	0.000*
Occupation^c^		
Professional	(Referent)	
Clerical	0.78 (0.72–0.84)	0.000*
Skilled agricultural	0.74 (0.69–0.79)	0.000*
Elementary worker	0.47 (0.43–0.51)	0.000*
Non worker	0.30 (0.28–0.32)	0.000*
Access to Media		
Good	(Referent)	
Fair	1.59 (1.53–1.65)	0.000*
Poor	2.39 (2.28–2.52)	0.000*
No access	2.09 (1.98–2.22)	0.000*
Tobacco Use^d^		
No	(Referent)	
Yes	1.04 (1.002–1.085)	0.041*
Attitude towards wife (among married respondents)^e^		
Good attitude	(Referent)	
Poor attitude	0.97 (0.88–1.06)	0.477
HIV/AIDS prevention knowledge^f^		
Yes	(Referent)	
No	0.83 (0.80–0.86)	0.000*

Note: 132 participants did not respond to circumcision questions

*p < 0.05

^a^One respondent have missing information for marital status

^b^2,567 participants have missing information for religion

^c^1,684 participants have missing information for occupation

^d^Five respondents have missing information for smoking

^e^Among the 32,256 married respondents, 1,423 (4 %) of them did not respond to questions on attitude towards wife

^f^For this variable, all participants (*N* = 12,806) was included. Of these, 1,896 said “don’t know” on questions for knowledge of prevention of HIV/AIDS

Unadjusted analysis of education attainment and MC status found correlation of higher education attained with higher odds of being circumcised for half of the countries included in this study. However, notable differences were observed for Malawi, Lesotho and Kenya where men with no education were more likely to be circumcised (Additional file 1: Table A1). The high likelihood of those with higher educational attainment and no education was also reflected when all countries were combined in our univariate analysis (Table 1). Tram et al. did not find significant correlation between MC status and education; however, only individual country data were analyzed. Tram et al.’s analysis on wealth quintile and place of residence also did not yield significant associations but our analysis showed that men in the richest quintile and resides in urban areas are both more likely to be circumcised [10].

We also explored the relationship between circumcision status and religious affiliation where we found that Muslim men are consistently more likely to be circumcised compared to others as well (Additional file 2: Table A2). The combined univariate analysis also shows Muslim men are much more likely to be circumcised than other religious groups. We did not include ethnicity as we want to explore more macro-level predictors and Tram et al. had already shown that ethnicity is a significant predictor of MC status [10].

This study explored certain variables that were not included in previous studies: marital status, occupation, access to media, tobacco use, attitude towards wife, total lifetime sexual partners, current number of wives/partners, age at first sex, safe sexual behavior, and HIV prevention knowledge by country and by region (combining country data sets). For most countries, married men are more likely to be circumcised (Table 2) which was also found in the unadjusted regional analysis (OR = 1.37, Table 1). Men who do not have “Professional” occupations are less likely to be circumcised in seven of the 11 countries and the combined analysis also showed lower odds of MC for non-professional men. Men’s level of access to media and its correlation to MC status across individual countries varied widely although the combined regional analysis showed that those with fair, poor and no access as more likely to be circumcised. Men who use tobacco are more likely to be circumcised in seven countries and the regional analysis showed similar result. No significant correlation was found between MC status and attitude towards wife. Six out of 11 countries showed that those who did not demonstrate HIV/AIDS prevention knowledge are less likely to be circumcised although Kenya showed the opposite correlation; other countries did not have significant relationships. Regionally, men who did not demonstrate sufficient HIV/AIDS prevention knowledge are less likely to be circumcised (OR = 0.83).

**Table 2:** Odds ratio of male circumcision status and socio-economic and behavioural characteristics across 11 priority countries

Country	Zimbabwe	Zambia	Tanzania	Swaziland	Rwanda	Namibia	Mozambique	Malawi	Lesotho	Kenya	Ethiopia
	*N* = 7,421	*N* = 6,495	*N* = 2,526	*N* = 4,155	*N* = 6,323	*N* = 3,912	*N* = 4,034	*N* = 7,159	*N* = 3,315	*N* = 3,464	*N* = 14,073
Marital Status											
Never in union	(Referent)	(Referent)	(Referent)	(Referent)	(Referent)	(Referent)	(Referent)	(Referent)	(Referent)	(Referent)	(Referent)
Married	1.70*	1.20*	0.88	2.27*	0.78*	1.39*	1.62*	1.17*	2.63*	1.51*	1.09
Separate	1.69*	1.32	1.19	2.46*	0.96	2.08*	1.34*	1.34	2.75*	1.96*	1.99*
Occupation^b^											
Professional	(Referent)	(Referent)	(Referent)	(Referent)	(Referent)	(Referent)	(Referent)	(Referent)	(Referent)	(Referent)	(Referent)
Clerical	1.13	0.98	0^a^	0.51*	0.48*	0.84	0.61*	1.72*	1.02	1.54	1.34
Skilled agricultural	0.89	1.06	0.26*	0.58*	0.11*	0.67*	0.68*	1	2.52*	0.66*	0.78
Elementary worker	0.92	0.88	0.66	0.44*	0.22*	0.72	0.57*	1.18	1.31	0.88	1.45
Non worker	0.67*	0.87	0.41*	0.26*	0.13*	0.53*	0.34*	0.89		0.39*	0.84
Access to Media											
Good	0^a^	(Referent)	(Referent)	(Referent)	0^a^	(Referent)	0^a^	(Referent)	(Referent)	(Referent)	0^a^
Fair	(Referent)	0.94	0.72*	0.73	(Referent)	0.70*	(Referent)	1	1.67*	0.75*	(Referent)
Poor	0.94	1.33*	0.66*	0.98	0.45*	0.69	1.78*	0.84	2.09*	1.05	0.50*
No access	0.90	1.2	0.26*	0.8	0.28*	0.51*	0.92	1.21	2.52*	1.05	0.38*
Tobacco Use^c^											
No	(Referent)	(Referent)	(Referent)	(Referent)	(Referent)	(Referent)	(Referent)	(Referent)	(Referent)	(Referent)	(Referent)
Yes	1.65*	1.29*	1.27	1.53*	0.8	1.37*	1.27*	1.07	2.20*	2.92*	0.83*
Attitude towards wife^d^											
Good attitude	(Referent)	(Referent)	(Referent)	(Referent)	(Referent)	(Referent)	(Referent)	(Referent)	(Referent)	(Referent)	(Referent)
Poor attitude	1.45	0.84	0.74	0.62	2.25	2.27	0.96	0.98	0.95	1.05	1.06
HIV/AIDS prevention knowledge^e^											
Yes	(Referent)	(Referent)	(Referent)	(Referent)	(Referent)	(Referent)	(Referent)	(Referent)	(Referent)	(Referent)	(Referent)
No	0.62*	0.76*	0.83	0.43*	0.53*	0.74*	0.96	1.07	1.11	1.26*	0.62*

Note: 0.02 % (Mozambique) to 0.79 % (Zimbabwe) participants did not respond to question on circumcision

*p < 0.05

^a^No respondents in this category

^b^0.02 % (Zimbabwe) to 30.4 % (Lesotho) participants have missing information for occupation

^c^0.02 % (Namibia) to 0.03 % (Malawi) participants have missing information for smoking

^d^This variable only includes married male respondents, of whom 0.43 % (Malawi) to 2.1 % (Kenya) did not respond to attitude towards wife

^e^1.5 % (Rwanda) to 11.6 % (Lesotho) participants said “don’t know” for HIV/AIDS prevention knowledge

For variables that we included on sexual behaviour, alcohol consumption at last sex did not have statistical correlation for most countries, except Kenya where there is positive relationship (OR = 1.72, Table 2). Interestingly, the regional analysis results showed a higher likelihood of circumcision (OR = 1.21) with alcohol consumption during last sex. Correlation of MC status and Safe Sexual Behaviour varied widely across different countries where Tanzania, Rwanda, Namibia, and Ethiopia showed a lower likelihood of MC for risky sexual behaviour while the opposite was found in Lesotho and Kenya. Overall, risky behaviour is correlated with MC regionally (OR = 1.65, Table 4). Number of total lifetime sexual partners is correlated with circumcision in eight countries where there is a higher likelihood of circumcision for those who have three or more partners (Table 3). In the regional analysis, however, men who have two or more partners are less likely to be circumcised (Table 4). In the attempt to understand participants’ current number of partners, we also included “Number of wives/partners currently” but no significant correlations were found. This could be due to the large proportion of missing information for this question where approximately 35 % responses were missing in the entire combined dataset. For most countries, statistically significant relationships were not found between Age at first Sex and MC status, except Namibia and Mozambique where those who reported having first sexual experience younger than 14 years of age were much likelier to be circumcised (OR = 3.42 and 10.1, respectively). Regionally, however, men who are circumcised are less likely to have had their first sexual encounter before 30 years of age. Finally, men who reported having STDs/STIs in the past 12 months are significantly less likely to be circumcised in Tanzania, Rwanda, Kenya and Ethiopia while the opposite is true for Namibia. Regional analysis showed that men who reported STI symptoms are less likely to be circumcised (OR = 0.64, 95 % CI = 0.58–0.69).

**Table 3:** Odds ratio of male circumcision status to sexual behaviour, as a subset analysis of men who reported to have had sex (ages 15+ years)

Country	Zimbabwe	Zambia	Tanzania	Swaziland	Rwanda	Namibia	Mozambique	Malawi	Lesotho	Kenya	Ethiopia
	*N* = 5,635	*N* = 5,587	*N* = 1,919	*N* = 2,930	*N* = 4,583	*N* = 3,370	*N* = 3,697	*N* = 6,156	*N* = 2,899	*N* = 2,926	*N* = 9,908
Alcohol consumption last time had sex^a^											
No	*Not in questionnaire*	(Referent)	*Not in questionnaire*	(Referent)	*Not in questionnaire*	(Referent)	*Not in questionnaire*	*Not in questionnaire*	(Referent)	(Referent)	(Referent)
Yes		0.83		1.37		1.03			1.20	1.72*	0.87
Safe Sexual Behaviour^b^											
Yes	(Referent)	(Referent)	(Referent)	(Referent)	(Referent)	(Referent)	(Referent)	(Referent)	(Referent)	(Referent)	(Referent)
No	1.05	1.19	0.54*	1.02	0.46*	0.78*	1.14	1.16	2.12*	1.83*	0.55*
Total lifetime sexual partners^c^											
1	(Referent)	(Referent)	(Referent)	(Referent)	(Referent)	(Referent)	(Referent)	(Referent)	(Referent)	(Referent)	(Referent)
2	1.06	1.32	0.72	1.18	1.05	1.41	1.47*	1.76*	0.95	1.14	1.00
3+	1.42*	1.84*	0.93	2.23*	1.71*	2.23*	3.32*	2.05*	1.45*	1.04	0.96
No. of wives/partners (currently)^d^											
1	(Referent)	(Referent)	(Referent)	(Referent)	(Referent)	(Referent)	(Referent)	(Referent)	(Referent)	(Referent)	(Referent)
> 1	0.85	1.14	0.89	1.3	0.65	1.84	0.82	1.13	1.67	0.38*	0.34*
Age at first sex^e^											
30+	(Referent)	(Referent)	(Referent)	(Referent)	(Referent)	(Referent)	(Referent)	(Referent)	(Referent)	(Referent)	(Referent)
25–29	0.70	1.69	0.26	1.09	1.01	1.79	2.12	0.60	1.42	1.14	0.64
20–24	0.74	1.46	0.11*	0.85	1.03	1.56	2.94	0.79	1.72	1.54	0.90
15–19	0.78	1.95	0.14	0.68	1.22	2.34	7.44	1.32	1.35	0.77	0.92
< 14	1.05	3.04	0.21	0.85	1.06	3.42*	10.1*	1.47	0.92	0.55	0.90
STDs/STIs in the last 12 months^f^											
No	(Referent)	(Referent)	(Referent)	(Referent)	(Referent)	(Referent)	(Referent)	(Referent)	(Referent)	(Referent)	(Referent)
Yes	1.18	1.05	0.49*	0.65	0.63*	1.68*	0.91	1.06	1.25	0.29*	0.45*

Note: Out of a total of 62,877 participants, 13,267 said they did not have sex. Hence, only the remaining N = 49,610 was included in this subset analysis of sexual behaviour

*p < 0.05

^a^11.3 % (Ethiopia) to 18.9 % (Namibia) has missing information for alcohol consumption during sex

^b^1.9 % (Mozambique) to 18.7 % (Namibia) participants has missing information on safe sexual behaviour

^c^0.53 % (Rwanda) to 12.9 % (Mozambiqu) participants said “don’t know” when asked for total lifetime partners

^d^20.4 % (Ethiopia) to 65.5 % (Swazilan) participants has missing information for No. of wives or partners currently

^e^0.21 % (Kenya) to 6.0 % (Lesotho) participants said “don’t know” for age at first sex

^f^0.11 % (Mozambique, Zambia) to 14.2 % (Swaziland) participants missing information for presence of STDs/STIs

**Table 4:** Odds ratio of male circumcision status to sexual behaviour, as a subset analysis of men who reported to have had sex, using all data combined for 11 countries

Characteristic	OR (95 % CI)	*p*-value
Alcohol consumption last time had sex^a^		
No	(Referent)	
Yes	1.21 (1.12–1.31)	0.000*
Safe Sexual Behaviour^b^		
Yes	(Referent)	
No	1.65 (1.58–1.73)	0.000*
Total lifetime sexual partners^c^		
1	(Referent)	
2	0.76 (0.72–0.81)	0.000*
3+	0.70 (0.67–0.74)	0.000*
No. of wives/partners^d^		
1	(Referent)	
> 1	1.03 (0.94–1.12)	0.573
Age at first sex^e^		
30+	(Referent)	
25–29	0.77 (0.65–0.89)	0.001*
20–24	0.62 (0.54–0.72)	0.000*
15–19	0.59 (0.51–0.68)	0.000*
< 14	0.58 (0.50–0.68)	0.000*
STDs/STIs^f^		
No	(Referent)	
Yes	0.64 (0.58–0.69)	0.000*

Note: Out of 62877 participants, 13267 said they did not have sex. Hence, 49,610 participants were included in the subset analysis on sexual behaviour

*p < 0.05

^a^25656 respondents said “don’t know” for alcohol consumption during sex

^b^6142 participants did not have responses on safe sexual behaviour

^c^2036 participants said “don’t know” when asked for total lifetime partners

^d^17304 participants did not have responses for number of wives or partners currently

^e^1340 participants said “don’t know” for age at first sex

^f^563 participants did not have information on presence of STDs/STIs

### Multiple logistic regression model

This model was constructed using multiple logistic regression with variables that are statistically significant in the regional unadjusted logistic regression. Excluded variables included Attitude towards wife and Number of wives or partners currently since they were not significant in the unadjusted analysis. Alcohol consumption at last sex was also excluded because 51 % of responses were not available as Zimbabwe, Tanzania, Rwanda, Mozambique and Malawi did not include this question in the questionnaire. Results of the multiple logistic regression showed that the estimate for each variable is adjusted for all other variables and therefore more reliable in terms of regional findings in comparison with unadjusted univariate analysis (Tables 4 and 5). Considering socio-economic correlates in the model, Place of residence (Rural), Religion (Christian and Others), Marital status (Married), Occupation (Clerical, Skilled agriculture, Elementary worker, Non worker), Wealth Index (Richer, Middle, Poorer, Poorest), and Tobacco use (Yes) all showed a lower likelihood of circumcision. Men who have no education, who have fair, poor or no access to media are more likely to be circumcised. Of these results, most were found to be similar compared to the regional unadjusted univariate analysis where lower odds of circumcision were associated with rural residence, Christian and Other religions, secondary education, non-professional occupations, richer to poorest, and fair to no access to media. Age is not significantly associated with MC status in the model while univariate analysis found that men who are older tend to be circumcised. Other differences were also found in marital status and tobacco use where the model showed that married men are less likely to be circumcised while univariate results showed higher likelihood; and men who use tobacco are less likely to be circumcised in the model while the opposite was found in the univariate analysis.

**Table 5:** Multiple logistic regression model

Characteristic		OR	(95 % CI)	*p*-value
Age		1.00	(0.99–1.01)	0.057
Residence *(Reference group = Urban)*				
	Rural	0.74	(0.69–0.79)	0.000*
Religion *(Reference group = Muslim)*				
	Christian	0.03	(0.02–0.03)	0.000*
	Others	0.01	(0.01–0.02)	0.000*
Marital status *(Reference group = Never in union)*				
	Married	0.84	(0.78–0.91)	0.000*
	Separated	1.10	(0.96–1.26)	0.159
Education *(Reference group = Higher)*				
	Secondary	0.63	(0.56–0.69)	0.000*
	Primary	0.96	(0.86–1.07)	0.491
	No education	2.18	(1.92–2.48)	0.000*
Occupation *(Reference group = Professional)*				
	Clerical/Sales	0.71	(0.63–0.80)	0.000*
	Skilled agriculture	0.71	(0.64–0.79)	0.000*
	Elementary worker	0.59	(0.52–0.66)	0.000*
	Non worker	0.35	(0.31–0.39)	0.000*
Wealth Index *(Reference group = Richest)*				
	Richer	0.55	(0.51–0.59)	0.000*
	Middle	0.58	(0.53–0.63)	0.000*
	Poorer	0.57	(0.52–0.62)	0.000*
	Poorest	0.46	(0.42–0.52)	0.000*
Access to Media *(Reference group = Good)*				
	Fair	1.32	(1.25–1.41)	0.000*
	Poor	2.13	(1.97–2.31)	0.000*
	No access	1.87	(1.69–2.06)	0.000*
Tobacco use *(Reference group = No)*				
	Yes	0.89	(0.85–0.96)	0.001*
HIV/AIDS prevention knowledge *(Reference group = Yes)*				
	No	0.93	(0.88–0.98)	0.005*
Safe Sexual Behaviour *(Reference group = Yes)*				
	No	1.17	(1.09–1.26)	0.000*
Total lifetime partners *(Reference group = 1)*				
	2	0.75	(0.69–0.82)	0.000*
	3+	0.86	(0.81–0.93)	0.000*
Age at first sex *(Reference group = 30+)*				
	25–29	0.96	(0.77–1.18)	0.096
	20–24	1.02	(0.83–1.25)	0.835
	15–19	1.19	(0.96–1.46)	0.096
	≤14	1.56	(1.25–1.93)	0.000*
STDs/STIs *(Reference group = No)*				
	Yes	0.68	(0.61–0.76)	0.000*

Odds ratio of circumcision status and participant characteristics (ORs are calculated by adjusting all variables with each other)

*p < 0.05

In terms of sexual behaviour, men who did not demonstrate knowledge of HIV/AIDS prevention, have two or more lifetime sexual partners and reported having STDs in the last 12 months are less likely to be circumcised. Men who show risky sexual behaviour and had sexual debut at 14 or younger are also more likely to be circumcised. Compared to unadjusted univariate analysis, model results for HIV/AIDS prevention knowledge, safe sexual behaviour, total lifetime partners and STDs/STIs were the same while age at first sex were different. Only those who reported sexual debut before 14 were found to be significantly more likely to be circumcised in the model (OR = 1.56, Table 5) while univariate analysis showed that men who reported age at first sex younger than 30 were all less likely to be circumcised (OR = 0.58–0.77, Table 4).

## Discussion

DHS Men’s questionnaires across 11 countries in the Southeastern African region were analyzed for correlates of MC and self-reported health outcomes. Since these questionnaires are standardized across populations and time, we analyzed individual country associations and also combined country data for regional analysis. In order to facilitate macro-level analysis, we have selected socio-economic and sexual behaviour factors that are similar amongst these countries and excluded variables that vary widely (e.g. ethnicity) for generalizability.

The association of MC status with age was consistent with previous studies in view of baseline circumcision prevalence where the likelihood of men being circumcised increases with age. In our multiple logistic regression model, the OR increases with age on a unit basis although the correlation was not statistically significant (*p* = 0.057). This finding should not be surprising as the age of traditional circumcision varies from birth to the 20s in different countries according to ethnic traditions [12]. Another similar finding was religious affiliation where Muslim men are significantly more likely to be circumcised when compared to other religions. Other studies have reported traditional circumcisions account for 30 % of global MC prevalence where two-thirds of these are among Muslims [12].

Although consistent results across different countries are often missing, certain trends in socio-economic associations with circumcisions did emerge. Overall, men with higher levels of educational attainment (secondary or higher), fall into the “Richest” wealth quintile, live in urban areas, and have “Professional” occupations are more likely to be circumcised. It seems that men in the higher socio-economic scale are also more likely to be circumcised. However, non-sexual health behaviour variables revealed conflicting results where men who use tobacco are more likely to be circumcised but circumcised men are also more likely to demonstrate HIV/AIDS prevention knowledge. All of these correlations hold true in our model except Marital status and Tobacco use where the regional univariate analysis and model results did not align. The reason for these inconsistencies is not known but may indicate the complexity of underlying trends and limitations of self-reported data.

Looking at sexual behaviour variables, it is interesting to see that circumcision is associated with risky sexual behaviour in the model although not in the unadjusted regional results. It must be noted that our assessment on safe sexual behaviour is largely based on condom use at last sex. Concerns have been raised on risk compensation associated with VMMC. However, a recent cohort study has shown compelling evidence of the lack of such an effect [13]. Risky behaviour have been shown to be correlated with concurrent partnerships and higher HIV prevalence regardless of MC status [3, 14]. Mishra and Bignami-Van Assche analyzed 22 DHS and AIDS Indicator surveys (AIS) across Africa and Southeast Asia (2001–2006) for association between self-reported concurrent relationships and HIV serostatus (adjusting for education, wealth, condom use, MC, etc.) at the individual, community and country levels [14]. This study found that those who are urban, wealthier and more-educated are more likely to be in concurrent relationships in the preceding 12 months. The authors also found that circumcised men are more likely to have concurrent partners. Although significant positive correlations were established for concurrent relationships with HIV prevalence at the individual level in SSA, this relationship did not hold at the community and country levels. One study explored factors associated with premarital and non-spousal sex. At the individual level, male with higher educational attainment and higher wealth status have higher odds of exhibiting high-risk behaviour [15]. Chikutsa et al. investigated the association of MC status and risky sexual behaviour in Zimbabwe using DHS 2010–2011 survey but found no association between MC and risky behaviour [16]. In Malawi polygyny was shown to correlate with HIV risk at the individual level where men are more likely to engage in extramarital sex than those who are in monogamous unions [17]. It is worth noting that such risk characteristics may be utilized for prioritizing circumcisions where the highest impact may be made in HIV/AIDS prevention strategies.

In our study, Age at first sex was not significantly associated with MC status, except for Namibia and Mozambique where sexual debut before 15 years was found to be correlated with circumcision. Mkwandawire et al. examined the association between timing of first sex and MC status using Malawi DHS performed in 2010 [18]. The study found that circumcision status is positively correlated with earlier sexual debut which may increase the risk of HIV infection [18]. As the VMMC program is being scaled up in different MC priority countries, this effect may be worthy of further research.

We have included qualitative measures on attitudes on gender relationships as reported by the male respondents by combining six questions under the combined variable of “Attitudes towards wife”. These questions are related to intimate partner violence (IPV) as studies have found elevated risk of poor sexual health outcomes, including HIV and STIs, by women subject to IPV [19–23]. One of these questions touched on negotiation power for condom use (“wife justified to ask husband to use condom if he has STD”) which also informs risks of infection. One study found that women who experienced abuse are 1.5 times less likely to ask their current partners to use condoms [24] while another study found that women with low relationship control are 2.1 times more likely to use condom inconsistently [25]. A recent study reported that couples who perceive their relationships more positively are associated with less risky sexual behaviour which is defined as more condom use and fewer partners [26]. However, IPV alone was not found to be associated with HIV prevalence and our study showed that men’s attitudes towards their partner were not correlated [27]. Of course, the questions included in this study were not posed to female partners of the men surveyed so the measurement is indirect. According to a recent global analysis of gender inequality and its impact on HIV transmission, there is significant correlation between heterosexual HIV epidemic and high gender inequality regardless of circumcision rate [28]. In addition, gender inequality was also found to be an important factor for the maintenance of generalized epidemics [28]. Since this study of baseline circumcision did not show statistical significant association with male circumcision, further research on the voluntary nature of the VMMC program may reveal evidence of gender power relations and circumcision choice. In our study, men who reported having had STD/STI symptoms were less likely to be circumcised. Although we were not able to link individual data to HIV status, the association between HIV/AIDS and STIs have been shown in different studies [29, 30]. In fact, it is reported that risk of contracting gonorrhea is lower among circumcised men [31] and chlamydia trachomatis infection among female partners of circumcised men is also lower [32].

As VMMC programs are being scaled up in different countries, it is important to consider how these findings may be used to achieve greater health impact. For example, in countries or communities where the wealthier, more educated men were positively correlated with MC status may serve as a positive image for social marketing strategies by leveraging existing social norms and counteracting cultural barriers [33]. In addition, we need to consider the prevalence of traditional circumcisions and potential adverse events (AE) that may entail [34]. In a systematic review of traditional circumcisions (i.e. not by medically trained providers) in eastern and southern African countries, of the six studies included, only two studies reported AE rates which are as high as 35 and 48 % [35]. Infection was cited as most frequent cause and the frequency of severe sequelae is generally higher following traditional circumcisions. In comparison, AEs result from circumcisions provided by trained professionals have shown to be much lower, where adult complication rates have cited as low as 2–4 %, but these rates vary depending on how adverse events are defined [12].

### Limitations

Variables used in this study are subjective health outcomes since they are self-reported via in-person interviews; hence, uncertainties in response accuracy are inherent [36]. Others have shown that accuracy of self-reported MC status can be problematic [37]. Furthermore, circumcision definitions may have different meaning where self-reported circumcision might be understood as attending “circumcision school or ceremony” where the portion of men actually circumcised may be small, as reported in Lesotho [12]. Incomplete circumcisions have also been reported to be prevalent [12]. We also found that responses were missing in nearly all variables and we have attempted to minimize the impact of missing data by excluding certain variables in our analysis. Lastly, we were not able to interpret on the potential impact of these findings on policy or programming decisions for MC programs in priority countries as the data used in this study were performed prior to MC scale-up.

## Conclusions

Certain correlations were found between male circumcision status and socio-economic and behaviour factors. These relationships vary at the country and regional levels. Our multiple logistic regression model found that men who are of the Muslim faith, reside in urban areas, have higher or secondary education attainment, hold professional occupations, and be in the richest wealth quintile are more likely to be circumcised. Circumcision is also positively correlated with lower reports of STIs, safe sexual behaviour, and HIV/AIDS prevention knowledge. However, inconsistencies were found for other variables that are difficult to explain. Since the circumcision data used for this study were collected prior to most country VMMC programs, further research is needed to elucidate the impact of VMMC scale-up and use the insights from baseline circumcisions to address cultural barriers that countries may face. Furthermore, a similar analysis using more up-to-date data from MC priority countries may provide insights useful for policy making and programmatic decisions.

## Additional files

Additional file 1: Table A1. List of combined variables with questions selected from the questionnaire, coding and definitions. This table does not contain data but additional information on some of the variables used in the study. (DOCX 18 kb) Additional file 2: Table A2. Odds ratio of male circumcision status and socio-economic and behavioural characteristics across 11 priority countries. This table contains the complete analysis results from Table 3. (DOC 165 kb)
