# Supplementary material for: Understanding the socio-economic and sexual behavioural correlates of male circumcision across eleven voluntary medical male circumcision priority countries in southeastern Africa
Source: BMC Public Health. 2015 Aug 22;15:813. doi: 10.1186/s12889-015-2135-1 (PMC4546248; doi:10.1186/s12889-015-2135-1)
Supplement: Additional file 1: Table A1. — List of combined variables with questions selected from the questionnaire, coding and definitions. This table does not contain data but additional information on some of the variables used in the study. (DOCX 18 kb) [file 12889_2015_2135_MOESM1_ESM.docx]

**Table A1**

**List of combined variables with questions selected from the questionnaire, coding and definitions.**

| **Combined Variable** | **Questions** | **Survey question number** | **Definition** |
| --- | --- | --- | --- |
| **Access to Mass media** | Frequency of reading newspaper or magazine | mv157 | Good Access: Respondent who watches television or listens to radio or reads newspaper almost everyday  Fair Access: Respondent who has not been included in the first category (good access) and who watches television or listens to radio or reads newspaper or magazines at least once a week  Poor Access: Respondent who has not been included in the first two categories (good or fair access to mass media) and who watches television or listens to radio or reads newspaper or magazines at least once a week.  No access: Respondent who never used any of the aforementioned mass media types |
|  | Frequency of listening radio | mv158 |  |
|  | Frequency of watching television | mv159 |  |
| **Attitude towards wife (married respondents only)** | Wife beating justified if she goes out without telling him | mv744a | Good: Respondent who answered “no” to all six questions.  Poor: Respondent who answered “yes” to any one of these questions. |
|  | Wife beating justified if she neglects the children | mv744b |  |
|  | Wife beating justified if she argues with him | mv744c |  |
|  | Wife beating justified if she refuses to have sex with him | mv744d |  |
|  | Wife beating justified if she burns the food | mv744e |  |
|  | Wife justified to ask husband to use condom if he has STD | mv822 |  |
| **Safe sexual behaviour** | Recent sexual activity | mv536 | Yes: Respondent who has never had sex or who uses condoms during last sex with most recent partner.  No: Respondent who is sexually active in the last four weeks and did not use condoms during last sex with most recent partner or respondent is not sexually active in the last four weeks and did not use condoms during last sex with most recent partner. |
|  | Condoms used during last time had sex with most recent partner | mv761 |  |
| **Sexually Transmitted diseases** | If yes, had genital sore/ulcer in the last 12 months | mv763b | Yes: Respondent who reports symptoms of either genital sore/ulcer or genital discharge in the preceding 12 months of the survey  No: Respondent who reports no history of the above symptoms in the preceding 12 months. |
|  | If yes, had genital discharge in the last 12 months | mv763c |  |
| **Knowledge of prevention of HIV/AIDS** | Have you been tested for HIV/AIDS | mv781 | Yes: Respondent who has been tested for HIV/AIDS and knows to always use condoms to reduce risk of HIV/AIDS.  No: Respondent who has not been tested for HIV/AIDS or does not know to always use condoms to reduce risk of HIV/AIDS or both. |
|  | Reduce the chance of AIDS by always using condoms | mv754cp |  |
| **Tobacco use** | Smokes cigarettes | mv463a | Yes: Respondent who smokes cigarettes or pipes or chews tobacco.  No: Respondent who does not smoke cigarettes, pipes and chews tobacco. |
|  | Smokes pipe | mv463b |  |
|  | Uses chewing tobacco | mv463c |  |
