# Supplementary material for: Understanding the socio-economic and sexual behavioural correlates of male circumcision across eleven voluntary medical male circumcision priority countries in southeastern Africa
Source: BMC Public Health. 2015 Aug 22;15:813. doi: 10.1186/s12889-015-2135-1 (PMC4546248; doi:10.1186/s12889-015-2135-1)
Supplement: Additional file 2: Table A2. — Odds ratio of male circumcision status and socio-economic and behavioural characteristics across 11 priority countries. This table contains the complete analysis results from Table 3. (DOC 165 kb) [file 12889_2015_2135_MOESM2_ESM.doc]

**Table A2**

**Odds ratio of male circumcision status and socio-economic and behavioural characteristics across 11 priority countries.**

| **Country** | **Zimbabwe** N=7,421  (n=696) | **Zambia**  N=6,495  (n=1040) | **Tanzania**  N=2,526  (n=2004) | **Swaziland**  N=4,155  (n=356) | **Rwanda**  N=6,323  (n=871) | **Namibia**  N=3,912  (n=764) | **Mozambique**  N=4,034  (n=1916) | **Malawi**  N=7,159  (n=1374) | **Lesotho**  N=3,315  (n=1854) | **Kenya**  N=3,464  (n=2913) | **Ethiopia**  N=14,073  (n=13004) |
| --- | --- | --- | --- | --- | --- | --- | --- | --- | --- | --- | --- |
| **Age groups, years** |  |  |  |  |  |  |  |  |  |  |  |
| 15-19 | 1.00 (referent) | 1.00 (referent) | 1.00 (referent) | 1.00 (referent) | 1.00 (referent) | 1.00 (referent) | 1.00 (referent) | 1.00 (referent) | 1.00 (referent) | 1.00 (referent) | 1.00 (referent) |
| 20-24 | 1.67*  (1.26 – 2.21) | 1.26*  (1.01 – 1.58) | 1.52*  (1.12 – 2.05) | 1.64*  (1.12 – 2.39) | 1.75*  (1.38 – 2.19) | 1.34*  (1.04 – 1.73) | 1.39*  (1.13 – 1.72) | 1.07  (0.89 – 1.28) | 4.27*  (3.43 – 5.32) | 2.87*  (2.14 – 3.84) | 1.65*  (1.36 – 2.02) |
| 25-29 | 2.08*  (1.59 – 2.73) | 1.11  (0.88 – 1.40) | 1.27  (0.92 – 1.74) | 2.01*  (1.36 – 2.99) | 1.87*  (1.48 – 2.37) | 1.39*  (1.07 – 1.80) | 1.66*  (1.34 – 2.06) | 0.91  (0.75 – 1.11) | 4.41*  (3.45 – 5.61) | 1.89*  (1.42 – 2.52) | 1.62*  (1.33 – 1.97) |
| 30-34 | 1.92*  (1.43 – 2.58) | 1.17  (0.92 – 1.47) | 1.36  (0.97 – 1.89) | 2.66*  (1.76 – 3.99) | 1.99*  (1.54 – 2.56) | 1.79*  (1.38 – 2.33) | 1.82*  (1.46 – 2.29) | 1  (0.82 – 1.23) | 3.83*  (2.96 – 4.95) | 2.37*  (1.75 – 3.21) | 1.81*  (1.44 – 2.27) |
| 35-39 | 2.47*  (1.85 – 3.31) | 1.50*  (1.18 – 1.91) | 1.34  (0.96 – 1.87) | 5.23*  (3.60 – 7.60) | 1.37*  (1.01 – 1.87) | 1.28  (0.95 – 1.74) | 2.11*  (1.67 – 2.67) | 1.08  (0.88 – 1.34) | 4.59*  (3.44 – 6.12) | 3.09*  (2.14 – 4.47) | 1.87*  (1.49 – 2.37) |
| 40-44 | 2.00*  (1.43 – 2.80) | 1.32  (0.99 – 1.75) | 1.21  (0.86 – 1.72) | 3.61*  (2.34 – 5.59) | 1.27  (0.92 – 1.77) | 1.56*  (1.14 – 2.13) | 1.86*  (1.42 – 2.44) | 1.08  (0.85 – 1.37) | 5.87*  (4.19 – 8.24) | 3.40*  (2.25 – 5.16) | 1.87*  (1.44 – 2.3) |
| 45-49 | 2.60*  (1.82 – 3.72) | 1.46*  (1.09 – 1.96) | 1.90*  (1.25 – 2.89) | 3.27*  (2.06 – 5.19) | 0.98  (0.68 – 1.42) | 1.37  (0.96 – 1.96) | 2.87*  (2.16 – 3.84) | 1.04  (0.81 – 1.34) | 3.92*  (2.84 – 5.41) | 2.70*  (1.79 – 4.06) | 1.70*  (1.29 – 2.24) |
| 50-54 | 2.29*  (1.58 – 3.34) | 1.37  (0.98 – 1.93) |  |  | 0.95  (0.65 – 1.39) |  | 1.87*  (1.38 – 2.54) | 1.15  (0.87 – 1.52) | 3.39*  (2.40 – 4.80) | 2.91*  (1.85 – 4.57) | 1.64*  (1.21 – 2.23) |
| 55-59 |  | 1.19  (0.79 – 1.79) |  |  | 0.59  (0.34 – 1.00) |  | 2.02*  (1.56 – 2.63) |  | 4.34*  (3.05 – 6.17) |  | 2.67*  (1.73 – 4.13) |
| **Place of residence** |  |  |  |  |  |  |  |  |  |  |  |
| Urban | 1.00 (referent) | 1.00 (referent) | 1.00 (referent) | 1.00 (referent) | 1.00 (referent) | 1.00 (referent) | 1.00 (referent) | 1.00 (referent) | 1.00 (referent) | 1.00 (referent) | 1.00 (referent) |
| Rural | 0.87  (0.74 – 1.02) | 1.09  (0.95 – 1.25) | 0.23*  (0.16 – 0.31) | 0.41*  (0.33 – 0.51) | 0.22*  (0.19 – 0.26) | 0.65*  (0.56 – 0.77) | 0.69*  (0.61 – 0.78) | 0.74*  (0.63 – 0.86) | 2.89*  (2.43 – 3.42) | 0.69*  (0.57 – 0.84) | 0.18*  (0.15 – 0.23) |
| **Marital Status** |  |  |  |  |  |  |  |  |  |  |  |
| Never in union | 1.00 (referent) | 1.00 (referent) | 1.00 (referent) | 1.00 (referent) | 1.00 (referent) | 1.00 (referent) | 1.00 (referent) | 1.00 (referent) | 1.00 (referent) | 1.00 (referent) | 1.00 (referent) |
| Married | 1.70*  (1.45 – 2.04) | 1.20*  (1.05 – 1.39) | 0.88  (0.72 – 1.07) | 2.27*  (1.81 – 2.84) | 0.78*  (0.67 – 0.89) | 1.39*  (1.18 – 1.65) | 1.62*  (1.42 – 1.86) | 1.17*  (1.03 – 1.32) | 2.63*  (2.27 – 3.05) | 1.51*  (1.25 – 1.81) | 1.09  (0.96 – 1.23) |
| Separate | 1.69*  (1.19 – 2.42) | 1.32  (0.97 – 1.78) | 1.19  (0.76 – 1.88) | 2.46*  (1.59 – 3.81) | 0.96  (0.61 – 1.49) | 2.08*  (1.49 – 2.92) | 1.34*  (1.02 – 1.77) | 1.34  (0.98 – 1.83) | 2.75*  (2.03 – 3.73) | 1.96*  (1.13 – 3.40) | 1.99*  (1.29 – 3.09) |
| **Education** |  |  |  |  |  |  |  |  |  |  |  |
| Higher | 1.00 (referent) | 1.00 (referent) | 1.00 (referent) | 1.00 (referent) | 1.00 (referent) | 1.00 (referent) | 1.00 (referent) | 1.00 (referent) | 1.00 (referent) | 1.00 (referent) | 1.00 (referent) |
| Secondary | 0.69*  (0.52 – 0.93) | 1.28  (0.88 – 1.87) | 1.22  (0.27 – 5.29) | 0.45*  (0.33 – 0.62 | 0.29*  (0.21 – 0.42) | 0.56*  (0.42 – 0.76) | 0.36*  (0.25 – 0.53) | 1.01  (0.67 – 1.53) | 1.03  (0.72 – 1.46) | 1.11  (0.79 – 1.57) | 0.80  (0.58 – 1.11) |
| Primary | 0.84  (0.62 – 1.15) | 1.02  (0.78 – 1.33) | 0.26  (0.06 – 1.10) | 0.37*  (0.27 – 0.53) | 0.08*  (0.06 – 0.11) | 0.44*  (0.32 – 0.61) | 0.29*  (0.21 – 0.44) | 1.49  (0.99 – 2.22) | 3.04*  (2.16 – 4.27) | 0.64*  (0.47 – 0.88) | 0.46*  (0.36 – 0.60) |
| No education | 0.88  (0.43 – 1.79) | 1.08  (0.83 – 1.41) | 0.15*  (0.03 – 0.66) | 0.52*  (0.33 – 0.83) | 0.06*  (0.04 – 0.09) | 0.79  (0.55 – 1.13) | 0.32*  (0.22 – 0.48) | 2.93*  (1.89 – 4.55) | 11.75*  (7.78 – 17.75) | 1.88*  (1.05 – 3.40) | 0.76  (0.57 – 1.00) |
| **Religion^i^** |  |  |  |  |  |  |  |  |  |  |  |
| Muslim | 1.00 (referent) | 1.00 (referent) |  | 1.00 (referent) | 1.00 (referent) | 0^†^ | 1.00 (referent) | 1.00 (referent) | 1.00 (referent) | 1.00 (referent) | 1.00 (referent) |
| Christian | 0.03*  (0.02 – 0.06) | 0.13*  *(0.06 – 0.29)* | *Not in questionnaire* | 0.24*  (0.06 – 0.92) | 0.05*  (0.03 – 0.08) | 1.00 (referent) | 0.11*  (0.09 – 0.14) | 0.007*  (0.005 – 0.01) | 0.70  (0.20 – 2.41) | 0.09*  (0.05 – 0.17) | 0.19*  (0.16 – 0.24) |
| Others | 0.03*  (0.01 – 0.05) | 0.30*  *(0.13 – 0.70)* |  | 0.26  (0.07 – 1.01) | 0.07*  (0.04 – 0.12) | 2.04*  (1.34 – 3.12) | 0.05*  (0.04 – 0.07) | 0.003*  (0.001 – 0.006) | 1.05  (0.29 – 3.68) | 0.18*  (0.08 – 0.41) | 0.03*  (0.02 – 0.05) |
| **Wealth Index** |  |  |  |  |  |  |  |  |  |  |  |
| Richest | 1.00 (referent) | 1.00 (referent) | 1.00 (referent) | 1.00 (referent) | 1.00 (referent) | 1.00 (referent) | 1.00 (referent) | 1.00 (referent) | 1.00 (referent) | 1.00 (referent) | 1.00 (referent) |
| Richer | 0.85  (0.68 – 1.06) | 1.27*  (1.03 – 1.57) | 0.23*  (0.14 – 0.35) | 0.60*  (0.45 – 0.80) | 0.26*  (0.21 – 0.31) | 0.79*  (0.61 – 0.99) | 0.55*  (0.46 – 0.65) | 1.03  (0.86 – 1.23) | 1.94*  (1.54 – 2.46) | 1.01  (0.76 – 1.33) | 0.29*  (0.23 – 0.38) |
| Middle | 0.68*  (0.53 – 0.87) | 1.59*  (1.29 – 1.96) | 0.14*  (0.09 – 0.22) | 0.39*  (0.28 – 0.55) | 0.19*  (0.16 – 0.25) | 0.94  (0.75 – 1.17) | 0.75*  (0.62 – 0.90) | 1.11  (0.93 – 1.32) | 3.21*  (2.56 – 4.04) | 0.93  (0.69 – 1.24) | 0.27*  (0.21 – 0.34) |
| Poorer | 0.78*  (0.61 – 0.99) | 2.18*  (1.75 – 2.72) | 0.08*  (0.06 – 0.13) | 0.46*  (0.32 – 0.66) | 0.16*  (0.12 – 0.21) | 0.55*  (0.41 – 0.73) | 0.82*  (0.68 – 0.99) | 1.20*  (1.00 – 1.44) | 4.34*  (3.45 – 5.46) | 0.56*  (0.43 – 0.74) | 0.21*  (0.17 – 0.28) |
| Poorest | 0.93  (0.74 – 1.18) | 1.34*  (1.07 – 1.68) | 0.11*  (0.07 – 0.16) | 0.38*  (0.26 – 0.57) | 0.15*  (0.11 – 0.20) | 0.34*  (0.24 – 0.47) | 0.72*  (0.59 – 0.88) | 0.91  (0.75 – 1.11) | 6.52*  (5.10 – 8.34) | 0.70*  (0.53 – 0.93) | 0.13*  (0.10 – 0.16) |
| **Occupation^ii^** |  |  |  |  |  |  |  |  |  |  |  |
| Professional | 1.00 (referent) | 1.00 (referent) | 1.00 (referent) | 1.00 (referent) | 1.00 (referent) | 1.00 (referent) | 1.00 (referent) | 1.00 (referent) | 1.00 (referent) | 1.00 (referent) | 1.00 (referent) |
| Clerical | 1.13  (0.76 – 1.67) | 0.98  (0.70 – 1.39) | 0^†^ | 0.51*  (0.31 – 0.82) | 0.48*  (0.34 – 0.68) | 0.84  (0.53 – 1.32) | 0.61*  (0.46 – 0.81) | 1.72*  (1.19 – 2.49) | 1.02  (0.63 – 1.63) | 1.54  (0.93 – 2.55) | 1.34  (0.95 – 1.91) |
| Skilled agricultural | 0.89  (0.64 – 1.26) | 1.06  (0.79 – 1.44) | 0.26*  (0.12 – 0.56) | 0.58*  (0.41 – 0.82) | 0.11*  (0.09 – 0.16) | 0.67*  (0.48 – 0.92) | 0.68*  (0.53 – 0.87) | 1  (0.72 – 1.41) | 5.87*  (4.19 – 8.24) | 0.66*  (0.49 – 0.86) | 0.78  (0.58 – 1.03) |
| elementary worker | 0.92  (0.64 – 1.32) | 0.88  (0.57 – 1.35) | 0.66  (0.28 – 1.49) | 0.44*  (0.29 – 0.67) | 0.22*  (0.16 – 0.29) | 0.72  (0.51 – 1.02) | 0.57*  (0.41 – 0.77) | 1.18  (0.81 – 1.71) | 1.31  (0.88 – 1.95) | 0.88  (0.64 – 0.86) | 1.45  (0.89 – 2.34) |
| Non worker | 0.67*  (0.47 – 0.95) | 0.87  (0.62 – 1.21) | 0.41*  (0.18 – 0.94) | 0.26*  (0.18 – 0.37) | 0.13*  (0.09 – 0.19) | 0.53*  (0.38 – 0.74) | 0.34*  (0.26 – 0.46) | 0.89  (0.61 – 1.30) |  | 0.39*  (0.29 – 0.54) | 0.84  (0.59 – 1.19) |
| **Access to Media** |  |  |  |  |  |  |  |  |  |  |  |
| Good | 0^†^ | 1.00 (referent) | 1.00 (referent) | 1.00 (referent) | 0^†^ | 1.00 (referent) | 0^†^ | 1.00 (referent) | 1.00 (referent) | 1.00 (referent) | 0^†^ |
| Fair | 1.00 (referent) | 0.94  (0.79 – 1.13) | 0.72*  (0.56 – 0.92) | 0.73  (0.49 – 1.06) | 1.00 (referent) | 0.70*  (0.55 – 0.89) | 1.00 (referent) | 1  (0.87 – 1.16) | 1.67*  (1.38 – 2.04) | 0.75*  (0.57 – 0.98) | 1.00 (referent) |
| Poor | 0.94  (0.76 – 1.14) | 1.33*  (1.08 – 1.64) | 0.66*  (0.47 – 0.92) | 0.98  (0.50 – 1.88) | 0.45*  (0.32 – 0.62) | 0.69  (0.49 – 1.00) | 1.78*  (1.37 – 2.31) | 0.84  (0.69 – 1.02) | 2.09*  (1.70 – 2.58) | 1.05  (0.67 – 1.63) | 0.50*  (0.44 – 0.58) |
| No access | 0.90  (0.72 – 1.14) | 1.2  (0.96 – 1.51) | 0.26*  (0.19 – 0.34) | 0.8  (0.44 – 1.46) | 0.28*  (0.12 – 0.63) | 0.51*  (0.33 – 0.79) | 0.92  (0.68 – 1.23) | 1.21  (0.96 – 1.54) | 2.52*  (2.09 – 3.05) | 1.05  (0.59 – 1.97) | 0.38*  (0.33 – 0.45) |
| **Tobacco Use^iii^** |  |  |  |  |  |  |  |  |  |  |  |
| No | 1.00 (referent) | 1.00 (referent) | 1.00 (referent) | 1.00 (referent) | 1.00 (referent) | 1.00 (referent) | 1.00 (referent) | 1.00 (referent) | 1.00 (referent) | 1.00 (referent) | 1.00 (referent) |
| Yes | 1.65*  (1.39 – 1.96) | 1.29*  (1.12 – 1.51) | 1.27  (0.97 – 1.64) | 1.53*  (1.16 – 2.03) | 0.8  (0.67 – 1.06) | 1.37*  (1.15 – 1.63) | 1.27*  (1.09 – 1.48) | 1.07  (0.92 – 1.24) | 2.20*  (1.89 – 2.56) | 2.92*  (2.14 – 3.97) | 0.83*  (0.71 – 0.99) |
| **Attitude towards wife^iv^** |  |  |  |  |  |  |  |  |  |  |  |
| Good attitude | 1.00 (referent) | 1.00 (referent) | 1.00 (referent) | 1.00 (referent) | 1.00 (referent) | 1.00 (referent) | 1.00 (referent) | 1.00 (referent) | 1.00 (referent) | 1.00 (referent) | 1.00 (referent) |
| Poor attitude | 1.45  (0.96 – 2.17) | 0.84  (0.58 – 1.19) | 0.74  (0.42 – 1.29) | 0.62  (0.25 – 1.54) | 2.25  (0.69– 7.27) | 2.27  (0.68 – 7.60) | 0.96  (0.77 – 1.19) | 0.98  (0.73 – 1.32) | 0.95  (0.59 – 1.54) | 1.05  (0.80 – 1.39) | 1.06  (0.74 – 1.52) |
| **HIV/AIDS prevention knowledge^v^** |  |  |  |  |  |  |  |  |  |  |  |
| Yes | 1.00 (referent) | 1.00 (referent) | 1.00 (referent) | 1.00 (referent) | 1.00 (referent) | 1.00 (referent) | 1.00 (referent) | 1.00 (referent) | 1.00 (referent) | 1.00 (referent) | 1.00 (referent) |
| No | 0.62*  (0.52 – 0.72) | 0.76*  (0.65 – 0.89) | 0.83  (0.67 – 1.03) | 0.43*  (0.34 – 0.55) | 0.53*  (0.44 – 0.63) | 0.74*  (0.62 – 0.87) | 0.96  (0.83 – 1.11) | 1.07  (0.95 – 1.21) | 1.11  (0.95 – 1.20) | 1.26*  (1.04 – 1.52) | 0.62*  (0.54 – 0.72) |

Note: n = number of circumcised men 15 years or older. 0.02% (Mozambique) to 0.79% (Zimbabwe) participants did not respond to circumcision.

*p<0.05

^†^No respondents in this category

^i^ 0.02% (Ethiopia, Malawi) to 0.38% (Namibia) participants has missing information for religion.

^ii^ 0.02% (Zimbabwe) to 30.4% (Lesotho) participants has missing information for occupation.

^iii^ 0.02% (Namibia) to 0.03% (Malawi) participants has missing information for smoking.

^iv^ This variable only includes married male respondents, of whom 0.43% (Malawi) to 2.1% (Kenya) did not respond to attitude towards wife.

^v^ 1.5% (Rwanda) to 11.6% (Lesotho) participants said 'don't know' for knowledge of prevention of HIV/AIDS.
